# Primary Cutaneous Atypical Spindle Cell Lipomatous Tumor

**DOI:** 10.1155/2021/4082289

**Published:** 2021-11-08

**Authors:** Madeline S. Tchack, Michael Broscius, Martin Reichel

**Affiliations:** ^1^Memorial Sloan Kettering Cancer Center, New York, NY 10065-6007, USA; ^2^Integrated Oncology, 521 West 57th Street, New York, NY 10019, USA; ^3^Department of Dermatology, College of Physicians & Surgeons, Columbia University, 161 Fort Washington Avenue, New York, NY 10032, USA

## Abstract

This report documents an exophytic, pedunculated nodule in a 74-year-old man that upon histopathological examination revealed an atypical spindle cell/pleomorphic lipomatous tumor (ASPLT) confined to the papillary and reticular dermis, representing the fourth documented case within the skin. Despite the overt pleomorphic changes present histologically, the patient is free of metastasis or recurrence five years after surgery.

## 1. Introduction

Primary neoplasms of fat within the papillary and reticular dermis occur sporadically. Pleomorphic liposarcoma, spindle cell/pleomorphic lipoma, and atypical lipomatous tumors have been documented [1–5]; however, only three previous examples of atypical spindle cell/pleomorphic lipomatous tumor (ASPLT) with dermal involvement have been reported [6, 7].

## 2. Case Presentation

A 74-year-old man presented with an erythematous pedunculated nodule measuring 1.5 cm in the greatest dimension on his right superior posterior thorax that had been present for at least two years prior. He was otherwise in good health, with no history of previous cutaneous or internal malignancy or remarkable dermatological history. Solar lentigines on the neck were the only other lesions noted at the time of examination. The patient had a shave biopsy and subsequently underwent wide excision and is now five years free of persistence at the primary site or evidence of metastasis.

**Figure 1:**
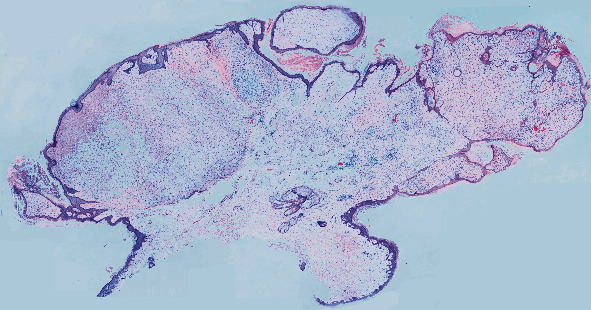
Scan shows a pedunculated neoplasm confined to the dermis that is composed of mixed areas of hypercellularity and hypocellularity. The stroma varies with predominantly myxoid areas and other foci that are more collagenous.

**Figure 2:**
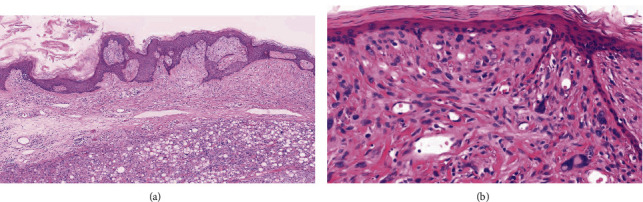
(a) Low power demonstrates a neoplasm abutting the epidermis composed of a hypocellular myxoid area overlying a more cellular area containing numerous univacuolar lipoblasts (H&E, 40x). (b) Sheets of spindle-shaped pleomorphic cells with enlarged hyperchromatic nuclei beneath the epidermis (H&E, 400x).

**Figure 3:**
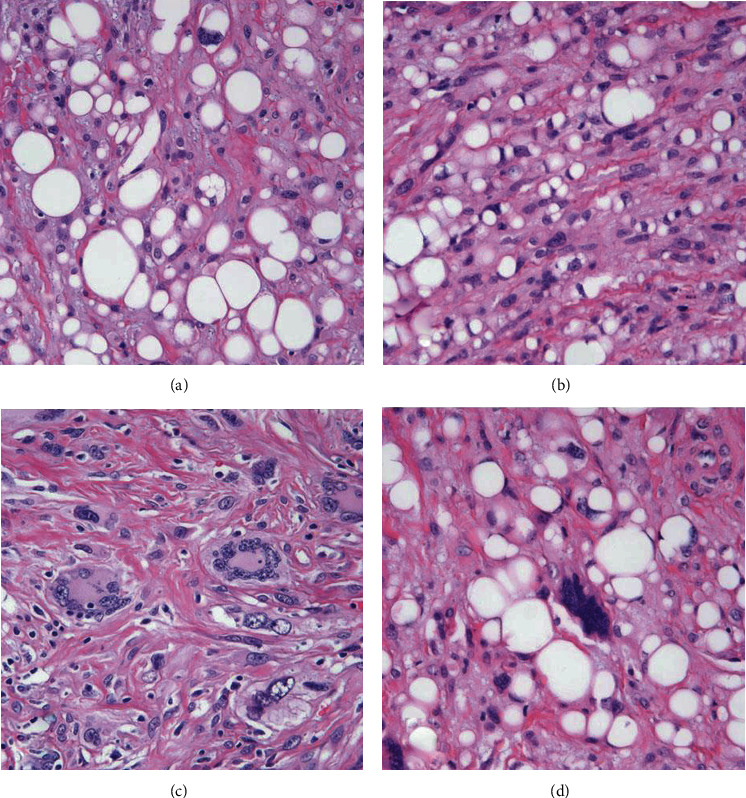
(a) Multivacuolated lipoblast surrounded by lipocytes that vary markedly in size and shape (H&E, 400x). (b) Area where univacuolar lipoblasts with scalloped nuclei predominate amid spindle cells with ovoid nuclei and the presence of ropey collagen (H&E, 400x). (c) Multinucleated giant cells with hints of ropey collagen (H&E, 400x). (d) Strikingly “bizarre” pleomorphic cell (H&E, 400x).

**Figure 4:**
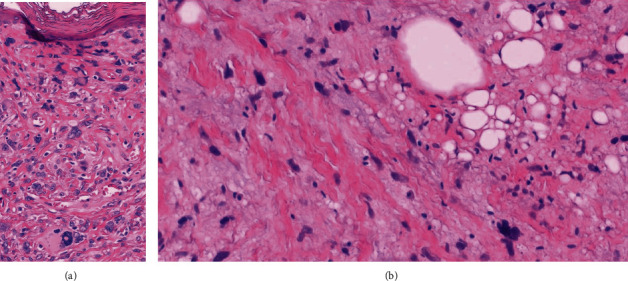
(a) Confluence of pleomorphic cells within the papillary dermis (H&E, 400x). (b) Thickened ropey collagen with myxoid stroma, pleomorphic cells, and variable lipoblasts (H&E, 400x).

**Figure 5:**
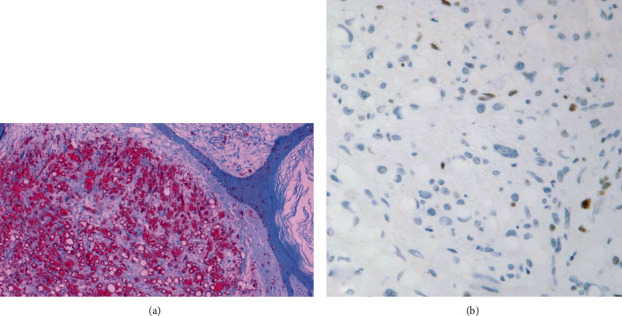
(a) S100 demonstrates prominent staining of univacuolar lipoblasts (100x). (b) Immunohistochemistry for Rb demonstrates absence of reactivity for Rb in the majority of tumor cells.

Histopathological examination features an exophytic, pedunculated intradermal neoplasm abutting the epidermis extending through the papillary and reticular dermis (Figure 1) without involvement of the subcutis. There is variable cellularity (Figure 2) composed of multiple cell types, including adipocytes with marked variation in size, spindle cells exhibiting nuclear atypia ranging from mild to severe (Figures 2(b), 3, and 4(a)), a few multivacuolated lipoblasts (Figure 3), focally abundant univacuolar lipoblasts (Figure 3(b)), and atypical multinucleated cells. Multinucleated giant cells are identified (Figure 3(c)) as well as areas of “ropey” collagen (Figure 4(b)). Rare mitotic figures are identified (<1/10 HPFs), necrosis is absent, and no pleomorphic lipoblasts are identified. The intercellular matrix is variable and includes both myxoid and collagenous areas. “Bizarre” pleomorphic cells are readily identified (Figure 3(d)). The neoplastic cells fail to display reactivity with immunostains for AE1-AE3, CD34, Desmin, MDM2, and CDK4, but in some small foci, labelling with S100, particularly among univacuolar lipoblasts, stain is present (Figure 5). There is significant loss of Rb expression (Figure 5(b)). Immunostains for HMB45 and MelanA are negative.

## 3. Discussion

Atypical spindle cell/pleomorphic lipomatous tumor (ASPLT) is a relatively recently described, benign adipocytic neoplasm that has been reported in a wide anatomic distribution, but occurs predominantly on the limbs [8]. ASPLT exhibits a disparate histologic appearance composed of varying degrees of mildly to moderately atypical spindle cells, lipoblasts, pleomorphic multinucleated cells, and matrix ranging from myxoid to collagenous. By immunohistochemical staining, they are often positive for CD34 but are typically either negative or only minimally immunoreactive for either MDM2 or CDK4, and MDM2 amplification by FISH is absent.

These lesions must be distinguished from other forms of adipocytic neoplasia. The presence of pleomorphic cells and lipoblasts suggests the more common intradermal pleomorphic liposarcoma; however, the rarity of mitotic figures, absence of necrosis, and lack of readily identifiable pleomorphic lipoblasts argue against the diagnosis of a pleomorphic liposarcoma. Both well-differentiated liposarcoma (atypical lipomatous tumor) and dedifferentiated liposarcoma are excluded by lack of either MDM2 or CDK4 staining. Spindle cell and pleomorphic forms of lipoma are excluded by the large number of lipoblasts and focal marked nuclear pleomorphism in the nonlipogenic areas. The presence of lipoblasts militates against both atypical fibroxanthoma and pleomorphic fibroma. Other than focal S100 expression, there are no histologic features of melanoma, and both HMB45 and MelanA immunohistochemical stains are negative. Lack of staining for AE1/AE3 excludes an epithelial neoplasm.

This patient had no evidence of recurrence or metastasis, further supporting the benign behavior of ASPLT. There are only three previously reported cases with cutaneous involvement [6, 7]. Although pleomorphic liposarcoma is the least common subtype of liposarcoma in man, pleomorphic liposarcoma is the most common subtype within the skin. It is important therefore to recognize ASPLT as a distinct neoplasm from pleomorphic liposarcoma.

Even though fat is not a normal inhabitant of the dermis, adipocytic neoplasms can arise intradermally and must be distinguished from both adipocytic and some nonadipocytic neoplasms of soft tissue origin presenting in the skin. The patient reported herein demonstrates that ASPLT appears to represent a unique neoplasm of the skin with adipose differentiation that, although exhibiting obviously pleomorphic histological features, appears to manifest an indolent clinical behavior.

## Data Availability

Data is available upon request.
